# Psychoactive Prescribing in Assisted Living

**DOI:** 10.1093/geroni/igaa057.2480

**Published:** 2020-12-16

**Authors:** Sheryl Zimmerman, Philip Sloane

## Abstract

More than 800,000 older adults reside in almost 30,000 assisted living (AL) communities across the country, 42% of who have moderate or severe dementia. As many as 97% of persons with dementia display symptoms of distress such as agitation or anxiety, and 69% of AL communities provide medications to treat this distress. Unfortunately, the psychoactive medications that are prescribed are often ineffective, contraindicated, and may cause serious adverse events including mortality. Especially concerning is the use of antipsychotic medications, which carry a black-box warning for persons with dementia. This symposium will present data from a seven state study of 250 AL communities and the 13,600 individuals who reside there. The first speaker will discuss the prevalence of psychoactive prescribing in AL overall and by medication type, and community characteristics that relate to use (e.g., staffing, resident case-mix). The second presentation will focus on the use of pro re nata (PRN) psychotropic medications, to examine the extent to which use is situational. The third speaker will address the use of off-label antipsychotic medications, and typologies of AL communities that differentiate use. The fourth speaker will discuss the prevalence of potential antipsychotic side-effects and adverse events, and also family member knowledge of medication use. The final speaker will compare the use of antipsychotic and antianxiety prescribing in proximate AL communities and nursing homes, to examine the extent to which local prescribing patterns may influence use. All five presentations of this symposium convey important issues for practice, policy, and future research.

